# Protection of Cattle against Epizootic Bovine Abortion (EBA) Using a Live *Pajaroellobacter abortibovis* Vaccine

**DOI:** 10.3390/vaccines10020335

**Published:** 2022-02-19

**Authors:** Myra T. Blanchard, Mike B. Teglas, Mark L. Anderson, Peter F. Moore, Bret R. McNabb, Kassidy M. Collins, Bret V. Yeargan, Jeffrey L. Stott

**Affiliations:** 1 Department of Pathology, Microbiology and Immunology, School of Veterinary Medicine, University of California-Davis, 1 Shields Ave, Davis, CA 95616, USA; mtblanchard@ucdavis.edu (M.T.B.); manderson@ucdavis.edu (M.L.A.); pfmoore@ucdavis.edu (P.F.M.); kmcollins@ucdavis.edu (K.M.C.); 2 Department of Agriculture, Veterinary and Rangeland Sciences, University of Nevada, 1664 N. Virginia St., Reno, NV 89557, USA; mteglas@unr.edu; 3 California Animal Health and Food Safety Laboratory, University of California, West Health Sciences Dr, Davis, CA 95616, USA; 4 Department of Population Health and Reproduction, School of Veterinary Medicine, University of CA—Davis, 1 Shields Ave, Davis, CA 95616, USA; brmcnabb@ucdavis.edu; 5 Department of Pathology and Laboratory Medicine, University of California-Davis Health, 2315 Stockton Blvd., Davis, CA 95817, USA; bvyeargan@ucdavis.edu

**Keywords:** epizootic bovine abortion (EBA), foothill abortion, *Pajaroellobacter abortibovis*, EBAA vaccine, early fetal losses, indirect fluorescent antibody test (IFAT), serology

## Abstract

Epizootic bovine abortion (EBA) is an arthropod-borne bacterial disease that causes significant economic loss for cattle producers in the western United States. The etiologic agent, *Pajaroellobacter abortibovis*, is an intracellular pathogen that has yet to be cultivated in vitro, thereby requiring novel methodologies for vaccine development. A vaccine candidate, using live *P. abortibovis*-infected cells (*P.a*-LIC) harvested from mouse spleens, was tested in beef cattle. Over the course of two safety studies and four efficacy trials, safety risks were evaluated, and dosage and potencies refined. No incidence of anaphylaxis, recognized health issues or significant impact upon conception rates were noted. Vaccination did result in subclinical skin reactions. Early fetal losses were noted in two trials and were significant when the vaccine was administered within 21 days prior to conception. Administration of the EBA agent (EBAA) vaccine as a single dose, at a potency of 500 *P.a*–LIC, 56 days prior to breeding, provided 100% protection with no early fetal losses. Seroconversion occurred in all animals following EBAA vaccination and corresponded well with protection of the fetus from epizootic bovine abortion.

## 1. Introduction

Epizootic bovine abortion (EBA; foothill abortion) is a vector-borne disease and is geographically limited by the distribution of the Argasid tick, *Ornithodoros coriaceus* Koch [1,2,3,4]; there are no other recognized methods of transmission. *Ornithodoros coriaceus*, commonly referred to as the Pajaroello tick, was first described in Mexico in the mid-1800s [5], with a distribution extending as far south as the state of Chiapas [6]. The full range of the vector, and thereby the disease, is not completely defined but epizootic bovine abortion is recognized in the dry foothill, mountainous and high desert regions of California, Oregon and Nevada [7,8], and while not reported in the literature, cattle in Mexico likely suffer EBA losses. Infected dams display no outward clinical signs of infection [7,9]. Devastating reproductive losses associated with the disease are primarily reported within the beef cattle industry as these animals are most often pastured in habitat that supports the tick vector, but losses can also occur in dairy cattle breeds [10,11]. Preventive measures have been limited to the management of vector exposure, either by pasturing cattle in known *O. coriaceus* habitat prior to breeding or by avoidance of tick infested habitats during pregnancy; neither are always efficacious or practical. Pre-breeding exposure of susceptible cattle to the tick vector can be helpful in establishing natural immunity to EBA, but success varies widely. *Ornithodoros coriaceus* feeds quickly, then drops back into the soil environment and may not feed again for months [12,13]. Vector activity is reduced in cold, rainy or windy conditions and thus opportunities for pre-breeding exposure in fall, winter and spring, particularly for replacement heifers, may be limited [8]. Furthermore, the percentage of ticks harboring the bacteria is typically <20%, and thus exposure to the bacterial pathogen, *Pajaroellobacter abortibovis,* is not guaranteed with each tick bite [3,4,14]. The economic impacts of EBA lie beyond tick vector borders as producers in EBA endemic areas hesitate to introduce cattle into their herds from non-endemic regions. Populations of naïve pregnant cattle, introduced into areas with *O. coriaceus* populations, can experience devastating fetal losses [15].

Epizootic bovine abortion is characterized by third-trimester abortion or birth of a full-term weak calf following an extended incubation period of >100 days [9,16,17]. No established connection between pre-breeding vector exposure and abortion has been identified, and therefore, vector avoidance prior to breeding has not been a consideration in EBA management strategies. Immunity in the dam is believed to be relatively long-lived, and an experimental challenge study demonstrated cattle are protected for a minimum of 1 year following abortion, with no evidence of strain variation [7,18].

Investigations into *P. abortibovis* have revealed an intracellular pathogen residing in macrophages [19,20]. The bacteria continue to elude attempts at in vitro cultivation, including cell culture [7,18,21]. The dramatic size reduction in the bacterial genome as compared to its closest known relative, *Sorangium cellulosum*, is consistent with bacterial linages transitioning from a free-living lifestyle into one characterized as being an obligate intracellular parasite [22]. Little is known about the relationship between *P. abortibovis* and the tick vector, aside from the bacteria being routinely identified within salivary glands. No association between tick infection and number of blood meals has been identified, bringing into question how the tick acquires *P. abortibovis* [4]. Members of the order Myxococcales, including *S. cellulosum*, are considered soil microbes [23]. Recent studies report 16S rRNA sequences from the genus *Pajaroellobacter* in cultivars of bahiagrass in southeastern USA [24] and in soil samples from the Tibetan Plateau [25], though it is unclear whether the sequences were identical to *P. abortibovis* or from unrecognized close relatives. The identification of *P. abortibovis* in soil would support the hypothesis that *O. coriaceus* acquires the bacteria directly from within its environment. Further research is needed to determine if this is a fastidious organism that could be grown in synthetic media once nutritional requirements are defined, or if *P. abortibovis* is truly an obligate intracellular bacterium.

The need for an efficacious vaccine to protect against EBA has existed since recognition of the disease in the 1950′s. Minimal knowledge of disease pathogenesis and the inability to propagate the bacteria using in vitro techniques has hampered vaccine design. The absence of demonstrable bacteria and/or clinical signs in experimentally infected cattle argued that development of a live vaccine represented a potential approach to disease control. However, a critical requirement for vaccine development is the establishment of potency, which is difficult without an in vitro cultivation system. Standard methodologies of bacterial quantitation, such as colony counts and optical densities of suspensions, do not apply to intracellular pathogens. Quantifying genes through PCR-based methodologies fails to address organism viability. Known sources of live *P. abortibovis* are limited to the infected bovine fetus and experimentally infected severe combined immunodeficient mice (SCID); the organism has not been identified in tissues from infected cattle nor is there evidence to suggest that *P. abortibovis* is capable of causing disease in any immunocompetent animal [7,17]. Obtaining sterile preparations of viable *P. abortibovis*-infected tissues from bovine fetuses that reliably transmit disease has proven difficult; fetal autolysis promotes microbial contamination, bacteria are opsonized, and bacterial loads vary widely from fetus to fetus [17,18,19,26]. Alternatively, spleen cells from *P. abortibovis*-infected SCID mice are relatively easy to harvest aseptically, appear to be consistently infectious, and bacteria are not opsonized [17]. The lack of apparent disease in infected mature cattle, evidence of long-term immunity following infection of cattle, and advances in both cryopreservation and flow cytometric methods to detect intracellular bacteria [20] encouraged researchers to explore the use of cryopreserved *P. abortibovis*-infected murine splenocytes as an effective vaccine for establishing immunity in naïve cattle prior to breeding. Data presented here are the first studies directed at developing a vaccine for preventing EBA.

## 2. Materials and Methods

### 2.1. Animal Sources and Maintenance

Studies to assess vaccine safety were conducted using heifers from the University of California, Davis (UCD) beef herd and housed at a facility in the Sacramento Valley. Cattle were either Angus or Hereford, biological type “*Bos Taurus*”, ranging from 10 to 22 months in age and co-mingled throughout each safety study. All were apparently healthy at the time of vaccination.

Vaccine efficacy trials were conducted using replacement heifers from the University of Nevada, Reno (UNR) beef cattle herd. Cattle were primarily Angus, with some *Bos indicus* hybrids (i.e., Brangus). Cattle were born, raised and maintained on irrigated pasture at the Main Station Field Laboratory (UNR-MSFL). Groups were co-mingled throughout the study period. At the time of the study, the tick vector had not been found on these premises despite several trapping attempts, nor had EBA been diagnosed in animals within the herd.

Cattle used in all efficacy trials were apparently healthy heifers with adequate size and conformation to serve as replacement heifers for UNR’s beef herd; selection of replacement heifers was made by UNR-MSFL management. Animal age at the time of vaccination ranged from 10 to 14 months in age. Criteria for exclusion prior to or following randomization included herd animals that (a) may have been exposed to *P. abortibovis* in utero over the course of previous bacterial challenge studies, (b) were suspected of having had prior natural exposure to *P. abortibovis* following serology studies, (c) were apparently ill at the time of initial vaccination or challenge, or (d) received antibiotic therapies for medical conditions that were not associated with the experiments.

### 2.2. Breeding, Pregnancy Evaluations and P. abortibovis Challenge Timelines

Cattle involved in efficacy trials were first synchronized and bred by artificial insemination (AI) over a 1-to-3-day period, rested between 18 and 24 days in order to allow non-pregnant animals to come back into estrus, and then exposed to bulls for 21 to 46 days of natural service (NS). Initial pregnancy evaluations were timed such that cattle bred by AI would be between 60 to 70 days of gestation. Veterinary staff conducted pregnancy examinations using a combination of transrectal palpation and ultrasound. Only cattle confirmed pregnant at the initial examination were candidates for participation in the efficacy portion of vaccine trials. Pregnancy examinations were again conducted at the time of challenge (described in Section 2.4). A minimum of one additional pregnancy examination for evaluation of early fetal loss was conducted in Trial #2, #3 and #4, prior to 100 days post-challenge (DPC) when fetuses were between 3 and 7 months of gestation. Heifers used in efficacy trials were divided into two bacterial challenge groups, such that the majority were challenged at 80 to 110 days of gestation [17,18,21]. In all but Trial #2, heifers bred by AI were challenged in the first group, and those bred by NS in the second. A detailed experimental timeline with associated data for each trial in this study is available in the Supplemental Materials section (Figure S1). Gestational ages were based upon estimated conception dates established during pregnancy examinations.

### 2.3. Vaccine Preparation and Testing

Epizootic Bovine Abortion Agent Vaccine, Live Culture, United States Department of Agriculture (USDA) product code, 1544.00 (unlicensed; EBAA, PC #1544.00) was used for all studies under the oversight of the USDA, Animal and Plant Health Inspection Service (APHIS), Veterinary Science, Center for Veterinary Biologics (CVB). The vaccine consisted of cryopreserved single-cell spleen suspensions derived from *P. abortibovis*-infected BALB/c *scid* mice (CBySmn.CB17-Prkdc scid/J), prepared and cryopreserved as previously described [20]. Briefly, spleens recovered from *P. abortibovis*-infected SCID mice were pressed through 100-µm Falcon nylon mesh cell strainers, washed once in Dulbecco’s phosphate-buffered saline (PBS) and cells pelleted at 300× *g* at 15 °C. Pellets were resuspended in cryopreservation media, aliquots rate frozen at −80 °C and transferred to liquid nitrogen within 48 h. Cells were stored in liquid nitrogen until use.

The *P. abortibovis* vaccine strain originated from two sources; (i) a naturally infected fetus, designated F92-0466, collected in Plumas County, California and (ii) an experimentally infected fetus, designated F99-0131, recovered after ticks collected in both Lassen County, California and Douglas County, Nevada fed on its dam. Bovine passages were accomplished by subcutaneous (SC) injection in the neck of cattle with *P. abortibovis*-infected homogenized fetal bovine thymus and/or spleen, cryopreserved in 20% glycerol at −80 °C as previously described [17]; homogenates from 1 to 4 fetuses were used for each passage inoculum. A total of 7 bovine passages were conducted; tissue homogenate from F99-0131 was combined with F92-0466-derived homogenates to generate dam inoculum for the 4th bovine tissue passage. All fetuses contributing *P. abortibovis*-infected tissues were collected between 105 and 124 days DPC and presented with microscopic lesions consistent with EBA [7,27].

The first murine passage was generated by inoculating BALB/c *scid* mice with cryopreserved, homogenized 7th passage fetal bovine thymus. Murine cryopreserved single-cell spleen suspensions were used for all subsequent passages and were administered by intraperitoneal route. Mice were euthanized between 56 and 73 DPC as they approached humane endpoints [20]. Spleens were harvested and vaccine prepared as described above. Vaccine serials for Trial #1 were generated from spleens harvested from the 1st sequential murine passage (P1) and Trial #2 from the 2nd (P2). One or both of two serials from the 6th murine passage (P6), designated P6-04-15 and P6-05-29, were used in the first and second safety study and in Efficacy Trial #3 and #4. Vaccine remained at liquid nitrogen temperature until thawed. Following thaw, cells were diluted in PBS to the desired potency and inoculated into the neck via SC route within 8 h. Cattle were observed for a minimum of 2 h post-vaccination for signs of anaphylaxis and observed at a close distance for signs of illness or injection site reactions for a minimum of 21 days.

Vaccine potency was based upon enumeration of live and infected murine splenocytes. The number of live cells/mL was determined using an ethidium bromide/acridine orange vital stain, diluted in PBS to final concentrations of 4 μg/mL each as previously described [28]. Analytical flow cytometry was applied to permeabilized, fixed cells incubated with fluorescence-conjugated anti-*P. abortibovis* antibodies to establish the percentage containing bacteria [20]. Potency was determined mathematically, multiplying the total # of live cells/mL by the percentage of *P. abortibovis*-infected cells to determine the number of live, *P. abortibovis*- infected spleen cells per dose (*P.a*.-LIC). Vaccine purity and safety were established using in vitro bacteriologic culture techniques as well as inoculation into immune-competent BALB/c mice and beef cattle (*Bos taurus*) of mixed breed per USDA CFR guidelines (9 CFR 113.27, 113.33 and 113.41, respectively). Placebo control groups were inoculated with vaccine diluent (i.e., PBS) as an alternative to preparations of spleens from uninfected SCID mice which contain few white blood cells [20]. The substitution was approved by USDA-APHIS-CVB.

### 2.4. Challenge Inocula

All efficacy trials included a challenge component. Pregnant cattle, between 71 and 112 days of gestation (DG), were inoculated in the neck by SC route with 1 mL of *P. abortibovis*-infected cell suspensions, derived from either fetal bovine or murine tissues. Challenge inoculum for Efficacy Trial #1 consisted of 0.5 gm fetal bovine spleen tissue homogenate cryopreserved in glycerol as described in Section 2.3, combined with single-cell suspensions of fetal bovine splenocytes at 50,000 *P.a.*-LIC; both sources of bovine tissue shared the same bacterial origin as the vaccine. Challenge inoculum for Trial #2 contained 40,000 *P.a.*-LIC single-cell suspension of pooled thymocytes and lymph node cells from naturally infected bovine fetus F09-13204, recovered in northern California; specific location information was not available. Challenge inoculum for Trial #3 contained 6000 *P.a.*-LIC of first passage murine spleen cells derived from bovine fetus F09-13204, and challenge inoculum for Trial #4 contained 1000 *P.a.*-LIC of first passage murine spleen cells originating from a naturally infected bovine fetus recovered in Yolo Co., CA. All single-cell tissue suspensions were prepared and cryopreserved in the same manner as the vaccine. Presence of *P. abortibovis* in all inoculum was confirmed by immunofluorescence [17]. The bovine fetal origin of challenge inocula used in Trial #2–4 differed from that of the vaccine, thereby providing a heterologous challenge if it was determined later that genetic and/or strain variants exist.

### 2.5. Safety Study #1: 21 Day Assessment

A preliminary study to assess vaccine safety within a 21-day period was conducted using six heifers, divided into 2 vaccine serial groups. Hair was shaved at injection sites for demarcation and repeated periodically throughout the study. Three heifers were inoculated with 2 mL of EBAA vaccine serial #P6-05-29 and three with 2 mL of #P6-04-15, each diluted 1:10 for final potencies of 8700 *P.a*.-LIC/mL and 6000 *P.a*.-LIC/mL, respectively. Animals were observed daily for changes in health status and injection site reactions, either visually at a close distance (days 1, 8, 9, 11–13 and 15–19 post-vaccination [DPV]) or by restraint in a squeeze chute for palpation of the injection site on 0, 2–7, 10, 14, 20 and 21 DPV. Reactions were measured (L × W in cm) and photographed. Deep muscle biopsies of injection sites were collected at 21 DPV. Similar biopsies were collected from the opposite side of the neck of two heifers for use as control sites.

### 2.6. Safety Study #2: 90 Day Assessment

Following assessment of results from Safety Study #1, which identified immune cell infiltrates at the injection site 21 DPV (refer to Section 3.1.2.), an extended study was conducted in a similar fashion to Study #1 but with several notable differences. Ten heifers were divided into two groups of five; one group received 1 mL of EBAA vaccine serial #P6-05-29 and the other received 1 mL of #P6-04-15, both diluted as described in Safety Study #1. Groups were treated one week apart for logistical purposes. One milliliter of placebo (i.e., diluent) was inoculated on opposite sides of the neck from vaccine as determined randomly by coin toss and blinded to observers. Cattle were restrained for health observations and injection site palpation 2 DPV and once weekly from 7 to 21 DPV, then twice weekly through the remainder of the 89-day observation period. Close observations without restraint were conducted all other days for the first 21 days and thereafter observations were made at time of feeding. Deep muscle biopsies of all injection sites were collected at 89 DPV unless otherwise noted.

### 2.7. Efficacy Trial #1: Potency and Dosage; 10,000 and 1000 P.a.-LIC

The first efficacy trial was a small preliminary study designed to explore the potential of using cryopreserved *P. abortibovis*-infected murine spleen cells as an effective method of vaccination. Potencies of 10,000 and 1000 *P.a*.-LIC were compared (Table 1). Twenty-two heifers were divided into 4 groups as follows. Group 1-A (n = 6) and 1-B (n = 5) were vaccinated with a P1 serial at a potency of 10,000 *P.a*.-LIC in either a single or 2-dose regimen, respectively (Table 1). One vaccinated heifer in Gr 1-A died prior to breeding, leaving five (refer to Section 3.1.1). Six additional heifers were vaccinated with 1000 *P.a*.-LIC of the same serial in a 2-dose regimen (Gr 1-C), and 5 heifers (Gr 1-D) received no injection (Table 1). Intervals between initial vaccination and initial breeding varied from 113 days for Gr 1-A and 1-B to 37 days for Gr 1-C (Table 1). Animals were not formally randomized nor were they blinded from observers. Injection sites were not demarcated. A total of 16 pregnant heifers, 4 from each group, were challenged with *P. abortibovis* (Table 2).

### 2.8. Efficacy Trial #2: Potency; 8000 and 1000 P.a.-LIC

Efficacy Trial #2 assessed potencies of 8000 and 1000 *P.a*.-LIC in a single-dose regimen from a P2 vaccine serial. Fifty-six heifers were divided into one of three groups: (2-A) 8000 *P.a*.-LIC, n = 28; (2-B) 1000 *P.a*.-LIC, n = 7; and (2-C) no vaccine, n = 21 (Table 1). Simple randomization, first by alternating groups and then breed of sires, was conducted to divide cattle into Gr 2-A and 2-C. Seven heifers were then selected out of the control group 2-C by no specific method after a retrospective change in design to create group 2-B. The study was not blinded to observers. All in Gr 2-B were bred to Angus sires. Breeding to Angus versus Brangus sires were staggered by 1 week for logistical purposes; intervals from vaccination to the initial date of AI are provided in Table 1. Injection sites were not demarcated nor observed for reactions. A total of 44 pregnant heifers were challenged with *P. abortibovis*: 25 from Gr 2-A, 7 from Gr 2-B and 12 from Gr 2-C (Table 2).

### 2.9. Efficacy Trial #3: Potency; 2000 P.a.-LIC

Efficacy Trial #3 tested safety and efficacy of EBAA vaccine at a potency of 2000 *P.a*.-LIC using 64 heifers divided equally into 2 single-dose groups: (3-A) 2000 *P.a*.-LIC of serial P6-04-15 and (3-B) Placebo (Table 1). Simple randomization of heifers into groups was accomplished by assigning a randomly generated number (Excel) to each cow’s ear tag number. The random numbers were sorted from low to high then divided in half, with treatment groups randomly assigned to each group by coin toss. The randomization, vaccine preparation and vaccine distribution were conducted by a single researcher and blinded from the other participants in the experiment. Hair was shaved to demarcate injection sites and repeated periodically throughout the observation period. Injection site reactions were monitored by palpation at 0, 7, 12, 19 and 21 DPV, along with a visual inspection of health status (e.g., cough, diarrhea, signs of malaise). A 21-day period elapsed between vaccination and breeding by AI (Table 1). A total of 44 pregnant heifers were challenged with *P. abortibovis:* 24 from Gr 3-A and 20 from Gr 3-B (Table 2).

### 2.10. Efficacy Trial #4: Minimum Effective Dose (500 P.a.-LIC) and Safety (500 and 2000 P.a.-LIC)

Efficacy Trial #4 was comprised of two components: (1) determine whether 500 *P.a*.-LIC was a sufficient potency to induce protection and thereby serve as the minimum effective dose for EBAA vaccine and (2) test vaccine safety for both 500 and 2000 *P.a*.-LIC potencies. Sixty-three heifers were divided into one of three groups by simple randomization in a manner similar to Trial #3 and given a single 1 mL injection of either EBAA vaccine serial P6-04-15 or PBS. Group design was as follows: (4-A) 2000 *P.a*.-LIC, n = 24; (4-B) 500 *P.a*.-LIC, n = 24; and (4-C) Placebo, n = 15. Five heifers were culled post-randomization by management and two others were removed post-vaccination after evidence of pre-trial *P. abortibovis* exposure (refer to Section 3.2.2), leaving group totals at 20, 22 and 14 for 4-A, 4-B and 4-C, respectively (Table 1). Vaccine preparation, distribution, blinding, injection site demarcation and daily animal health observations were conducted in the same manner as described in Trial #3. Injection site examinations were performed at 0, 20, 35, 47 and 54 DPV, in the same manner as previously described. The interval between vaccination and breeding by AI was 56 days (Table 1). A total of 34 pregnant heifers were challenged with *P. abortibovis*: 5 from Gr 4-A, 19 from 4-B and 10 from 4-C (Table 2). One of the ten challenged heifers from Gr 4-C was euthanized 34 DPC due to a chronic foot infection, leaving 9 placebo controls in the efficacy phase of the trial.

### 2.11. Histologic Studies of Injection Site Biopsies

Tissue biopsies were submitted to the California Animal Health and Food Safety (CAHFS) Laboratory System for formalin fixation, paraffin embedding, thin sectioning and Hematoxylin and Eosin (H&E) staining by standard methodologies. Stained tissue sections were evaluated by a board certified (ACVP) veterinary pathologist blinded to vaccine group identification.

### 2.12. Serologic Test

Blood was collected from cattle in serum separator tubes via either the jugular or tail vein. Serum was harvested following centrifugation and stored at −20 °C until analyzed. Blood samples for Trial #3 were collected from heifers at the time of vaccination (0 DPV; n = 64) and on day of challenge (119 or 147 DPV; n = 44). Samples were also collected from heifers in Trial #4 at 0 DPV (n = 58) and on day of challenge at either 146 or 181 DPV (n = 34). An indirect fluorescent antibody test (IFAT) was conducted as previously described [29]. Briefly, single-cell suspensions of *P. abortibovis*-infected SCID mice splenocytes, adjusted to a final concentration of 3.3 × 10^7^ cells/mL with PBS, were dispersed evenly over each well surface of 12-well Teflon-coated slides, fixed in acetone, air-dried and stored at −20 °C. Fluid samples were serially diluted with PBS, added to antigen-coated wells and incubated at room temperature for 20 min. Fluids were removed, slides were washed in PBS and goat anti-bovine IgG (heavy + light chains) conjugated to DyLight 488 was added to each well. Slides were incubated at room temperature for 15 min, washed as before, coverslips mounted, and wells viewed under a fluorescent microscope. Fetal fluids were initially diluted 1:10 with a maximum of 4 × 10-fold dilutions and cow sera diluted 1:200 followed by a maximum of 4 × 2-fold serial dilutions. Serology studies on cow sera were conducted after experiments were completed.

### 2.13. Early Fetal Losses

Cattle were classified as having experienced an early fetal loss if they were determined to be pregnant in the 1st trimester of gestation and open in a subsequent pregnancy exam.

### 2.14. Calving Outcomes

Pregnant cattle advancing to the efficacy portion of trials were observed daily for signs of abortion beginning 90 DPC. Fetuses and dead neonates, when found, were submitted to CAHFS diagnostic laboratory for necropsy. Weak, unhealthy calves were humanely euthanized if deemed necessary. Abortion panels were conducted to identify fetal/neonatal losses due to other abortifactive agents. Diagnostic analysis included culture for aerobic bacteria, *Campylobacter* spp. and *Brucella* spp. as well as direct fluorescent antibody tests to identify the presence of *Leptospira* species (kidney), BHV-1 and BVDV (kidney and lung). Fetal fluids were tested for antibodies directed against bovine herpesvirus-1 (infectious bovine rhinotracheitis; IBR), bovine viral diarrhea virus (BVDv), *Neospora caninum* and *Leptospira* spp. Assays to support EBA diagnoses included macro- and microscopic lesions [7,27] and elevated serum IgG [29,30] as well as *P. abortibovis*-specific immunohistochemistry (IHC; [19]), duplex PCR [26] and IFAT [29].

Positive fetuses were defined as those with lesions consistent with, or suggestive of EBA, in combination with elevated serum IgG and IFAT titers ≥ 1000. Detection of *P. abortibovis* by either PCR or IHC was desired but only required to confirm diagnosis if histopathology was inconclusive and/or if fluid for serology was either unattainable, the calf had fed on colostrum or IFAT titers were <1000. Dams found open after the last pregnancy exam with or without documentation of abortion were classified as undetermined losses, as were fetuses with equivocal EBA testing results (i.e., those meeting some but not all of the criteria for EBA diagnosis). Calves born apparently healthy were considered EBA-negative, as were fetuses diagnosed with alternate etiologies.

### 2.15. Study Outcomes

Outcomes related to safety included the risk of (a) developing anaphylaxis post-vaccination (b) becoming ill within a 90-day post-vaccination period, (c) developing injection site reactions, (d) vaccination interfering with conception, and (e) pregnant dams losing their fetus between 3 and 7 months of gestation. The primary outcome in determining vaccine efficacy was disease prevention in calves born to vaccinated dams that had been challenged with *P. abortibovis*. The immune status of heifers to *P. abortibovis* was evaluated by serologic methods (i.e., IFAT assay) to help assess pre-study exposure and immune status post-vaccination.

### 2.16. Statistical Analysis

Relative risk (RR) of vaccination resulting in development of an injection site reaction, a reduced conception rate or an increase in early fetal mortality as well as predictive values associated with serologic data were analyzed using 2 × 2 contingency tables and 95% confidence intervals (CIs) computed with Koopman asymptotic score. P values were calculated using a Fisher’s exact test. Prevented fraction (PF) values and 95% CIs were calculated as 1-RR [31] and used to evaluate vaccine efficacy as a function of both (a) calves developing EBA and (b) calf losses where EBA could not be ruled out (i.e., diagnosed EBA plus undetermined causes). All analysis was conducted using GraphPad Prism for Windows, version 9 (GraphPad Software, La Jolla, CA, USA). Notations of significant differences refers to *p* < 0.05 unless otherwise noted.

## 3. Results

### 3.1. Outcomes—Vaccine Safety

#### 3.1.1. Animal Health

No evidence of vaccine-associated immediate adverse effects, including anaphylaxis, were noted in any of the six studies reported here. All animals in all studies remained apparently healthy for a minimum of 21 DPV. Heifers in Safety Study #2 remained apparently healthy throughout the 89-day duration of the experiment, with the exception of one animal noted as lame at 45 DPV. Despite medical intervention, lameness progressed, and she was humanely euthanized at 67 DPV, at which time biopsies from the injection site were collected and processed as described in Section 2.11. No illnesses were noted in cattle from efficacy trials within the first 90 DPV. One heifer in Gr 1-A died abruptly at 110 DPV; there was no apparent association with vaccination but cause of death was not determined. One heifer in the placebo group of Efficacy Trial #3 (Gr 3-A) became severely lame post challenge (PC), was treated with antibiotics and supportive care, but required euthanasia at 180 DPV (34 DPC). The lameness was attributed to septic arthritis and veterinary staff humanely euthanized the animal; a necropsy was not performed.

#### 3.1.2. Injection Site Reactions

**Figure 1:**
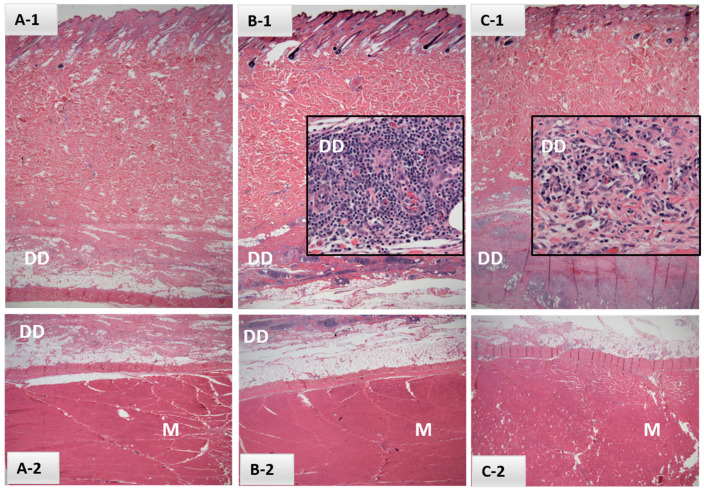
Histological sections of dermis (**A**-**1**,**B**-**1**,**C**-**1**), deep dermis (DD; **A**-**1**,**B**-**1**,**C**-**1** and **A**-**2**,**B**-**2**) and underlying muscle (M; **A**-**2**,**B**-**2**,**C**-**2**) from samples collected in Safety Study #1 from either control (**A**) or EBAA vaccine injection sites (**B**,**C**) at 21 days post-inoculation (H&E, 2×). Intervals between last palpable lesions and sample collection were (**B**) 14 days and (**C**) 1 day. Inserts (**B**-**1**,**C**-**1**) focus on perivascular mononuclear inflammatory cell infiltrates within the DD, consisting primarily of lymphocytes and plasma cells (40×).

Palpable injection site reactions were noted in two of the six heifers in Safety Study #1; one first noted at 2 DPV with a 5-day duration and the other noted first at 20 DPV and again at 21 DPV. Reactions ranged in size from 2.0 to 12.5 mm^2^. Localized inflammation was limited to swelling with no abscesses, purulent discharge, bleeding, ulcerations, granulomas or alopecia and no indication of heat or pain at the injection sites. Perivascular mononuclear inflammatory cell infiltrates, consisting largely of lymphocytes and plasma cells, were noted in the deep dermis adjacent to the panniculus muscle in sections taken from EBAA vaccination sites of both affected heifers at 21 DPV (Figure 1 B-1, C-1). Superficial and deep dermis sections taken from injection sites of the four remaining heifers and the two control sites appeared normal. Skeletal muscle appeared normal in all eight biopsy sites.

**Figure 2:**
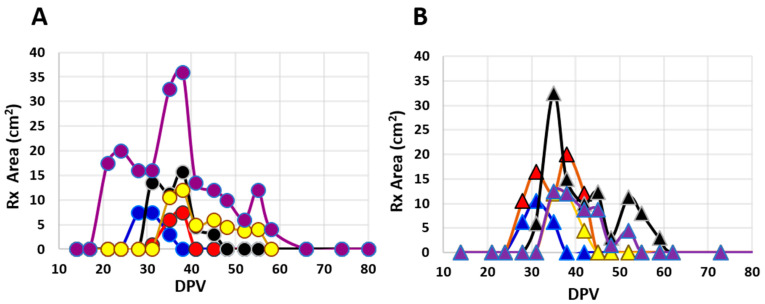
Areas (L × W) of palpable injection site reactions over time (days post-vaccination; DPV) in individual cattle from Safety Study #2 following inoculation with one of two EBAA vaccine serials. Potencies of serials were (**A**) 8700 or (**B**) 6000 *P.a.*-LIC with group sizes of five animals each.

**Figure 3:**
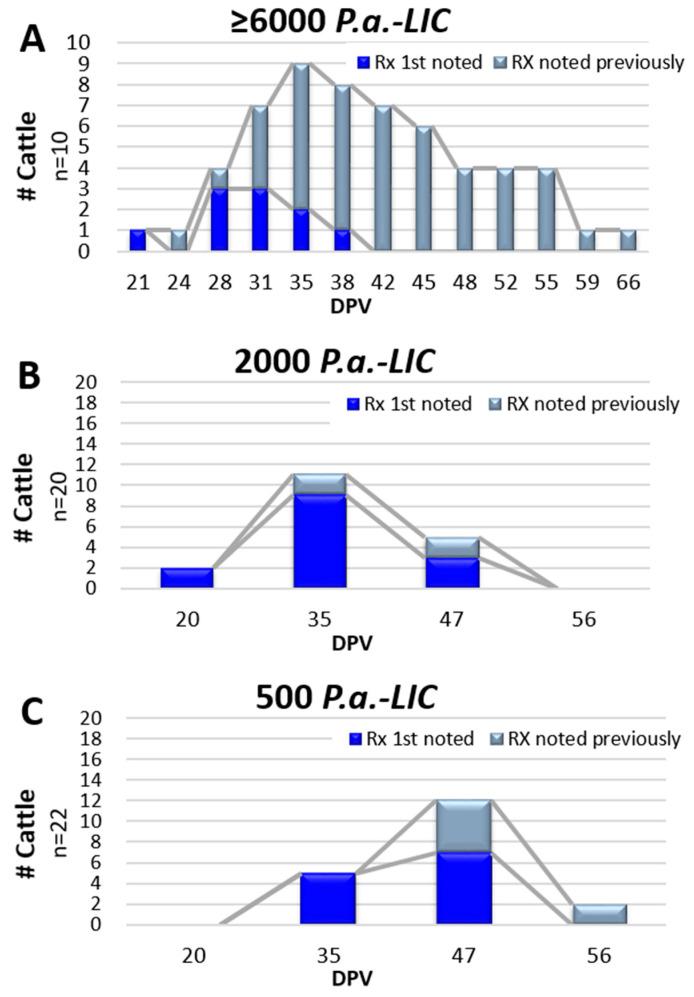
Temporal appearance of injection site reactions (Rx) following inoculation of EBAA vaccine. Vaccine potency varied and is provided at the top of each graph. Data from Graph (**A**) was derived from Safety Study #2 while data from Graphs (**B**,**C**) were derived from Efficacy Trial #4. First-time reactions in individual animals are depicted in blue and persisting reactions depicted in gray.

Palpable reactions in Safety Study #2 were noted in all 10 heifers at EBAA injection sites as compared to no reactions at placebo injection sites in control heifers; the difference was significant (RR = ∞; 95% CI: 2.0–∞). Reactions varied widely in size and duration (Figure 2); many were subtle and identifiable only by palpation. Discernable reactions were consistent with those noted in Safety Study #1 and areas of swelling ranged from 1.0 mm^2^ to 36.0 mm^2^, with a mean of 10.6 ± 7.3 mm^2^. Reactions were first noted between 21 and 38 DPV with duration ranging from 7 to 45 days (mean = 16.6 ± 12.2, median = 14) (Figure 2 and Figure 3A). All injection-site tissue biopsies collected at 89 DPV appeared normal, both grossly and histologically.

Injection site observations were made at four time points in Trial #3 at 7, 12, 19 and 21 DPV. Reactions, similar to those described above, were noted in 6.25% (2 of 32) of the vaccinated heifers (Gr 3-A) on 7 and/or 12 DPV and in 12.5% (4 of 32) of heifers given placebo (Gr 3-B) at 7, 12, 19 and/or 21 DPV (RR = 0.500; 95% CI: 0.122–2.18); the difference was not significant.

Injection site observations in Trial #4 were conducted at four time points; 20, 35, 47 and 56 DPV. Palpable reactions were noted in 61.9% (26 of 42) of the vaccinated heifers (14 of 20 in Gr 4-A and 12 of 22 in 4-B) and one heifer in placebo Gr 4-C (6.7%). The risk of developing an injection site reaction in the vaccinated animals was significant; RR = 8.79; 95% CI: 1.94–49.8. Results were similar when analyzed by the individual vaccine groups compared to the placebo group, but no difference was observed when comparing vaccinated Gr 4-A to Gr 4-B (RR = 1.1; 95% CI: 0.70–1.84). Reactions were consistent with previous studies. Areas of swelling in Gr 4-A ranged from 1.5 mm^2^ to 20.0 mm^2^, with a mean of 8.1 ± 4.5 mm^2^ and those in Gr 4-B ranged from 1.0 mm^2^ to 24.0 mm^2^, with a mean of 10.0 ± 6.5 mm^2^. Reactions were noted at 20, 35 and 47 DPV in Gr 4-A, with the majority (78.6%) noted only once (Figure 3B). By comparison, initial reaction observations in those given the lower potency of 500 *P.a.*-LIC (Gr 3-B) were noted at 35 and 47 DPV with reactions persisting in two heifers at 56 DPV (Figure 3C). Statistical comparisons as to whether vaccine potency influenced duration between vaccination and initial reaction observations, or duration of palpable reactions were hampered by limited data point collections.

#### 3.1.3. Risk of Decreased Conception

Conception rates in the efficacy trials ranged from 80% to 100%. Rates were equal to or higher in EBAA vaccinates in Trial #1 and #2 compared to the placebo controls but lower in Trial #3 and #4 (Table 1). Risk (RR) of vaccinates experiencing decreased conception rates in Trial #s 1–3 ranged from 0 to 1.67. Differences were not significant regardless of trial or group (Table 1), nor was there a significant difference in conception rates if data from all four trials were combined (RR = 1.49; 95% CI: 0.581–3.90).

Data was also analyzed by method of conception (Table 1). There were no significant differences between EBAA vaccinates and placebo control groups as to whether dams conceived by AI or NS, regardless of group, trial or if data from all four trials were combined and analyzed for all vaccinates versus all controls.

#### 3.1.4. Risk of Early Fetal Losses

Early fetal losses were noted in 20% of *P. abortibovis*-challenged, EBAA vaccinated heifers in Trail #2, group A and 41.7% of challenged, vaccinated heifers in Trial #3, group A (Table 2). Losses in Gr 2-A were distributed between the 1st and 2nd challenge groups (n = 3 and 2, respectively); intervals between vaccination and conception date ranged from 88 to 109 days. Breed of sires varied; the three losses in the 1st challenge group were artificially inseminated with Angus semen and the two in the 2nd challenge losses were bred to Brangus bulls by natural service. Relative risk equaled infinity due to an absence of losses in the placebo control group, but the difference was not significant (Table 2). When analyzed by challenge groups, results were similar. In contrast, all early fetal losses in Gr 3-A occurred in AI-bred animals that conceived 21 DPV (challenge group 1) and represented a 47.6% loss within that breeding/challenge subgroup. The difference between early fetal losses in challenged heifers from EBAA vaccinated (Gr 3-A) versus placebo control groups (Gr 3-B) was significant (Table 2).

### 3.2. Outcomes—Vaccine Efficacy

#### 3.2.1. Disease Prevention

A total of 34 abortions due to EBA occurred in challenged heifers over the course of the four efficacy trials (Table 3); all within placebo control groups. No additional abortifactive agents were identified. The incubation period between challenge and abortion ranged from 102 to 157 days with gestational ages at time of abortion between 191 and 262 days (see Figure S1).

Healthy calves made up the majority of those categorized as EBA-negative. Estimated gestational ages at birth of healthy calves ranged from 265 to 333 days (see Figure S1). The single exception was a full-term loss at 276 days of gestation in Gr 3-A; all diagnostic tests for EBA were negative (Table 3).

Most losses categorized as “undetermined” were cattle identified to be open late in gestation. Exceptions included two weak calves that subsequently died. One weak calf from a dam in challenge group 1 of Gr 3-B was born premature at 237 days of gestation (139 DPC). The calf died at 14 days of age. Necropsy revealed bronchopneumonia with enteritis, but no pathogenic organisms were identified. Underlying etiology was suspected to be EBA but *P. abortibovis* was not detected in tissues by either PCR or IHC. The other was a full-term calf (185 DPC at 282 days gestation) from challenge group 2 of Gr 4-B with no gross indications of EBA; tissues were not submitted for diagnostic analysis. In both cases, ingestion of colostrum negated the utility of serum IgG concentration or *P. abortibovis* serology as diagnostic aids.

Epizootic bovine abortion agent vaccine proved 100% effective in preventing diagnosed EBA regardless of potency. Differences between vaccinates and placebo controls were significant in Trial #2 and #4 regardless of whether data was analyzed by total number of animals challenged or challenge groups. Differences were significant in Trial #3 when data was analyzed by total number of animals challenged and those in challenge group 1, but not in challenge group 2 (Table 3) due to low numbers of data points. Broadening the definition of disease prevention to include calf losses in cases where EBA could not be ruled out as causation (i.e., EBA plus undetermined causes) reduced prevented fractions in Gr 2-A, Gr 3-A and Gr 4-B to 0.85, 0.64 and 0.95, respectively; results were significant. When calculated by challenge group, data remained significant for all but challenge group 2 within Trial #3, Gr 3-A (Table 3).

#### 3.2.2. Serology

Sera collected at the initiation of Trial #3 were tested for the presence of *P. abortibovis* antibody; all titers were <200 (n = 64). When pre-challenge sera (119/147 DPV) were tested, all challenged, vaccinated animals (Gr 3-A; n = 24) had seroconverted (titers ≥ 1600) while titers for challenged, unvaccinated controls (Gr 3-B; n = 20) remained undetectable.

Sera collected at the initiation of Trial #4 were negative for *P. abortibovis* antibody in 56 of 58 heifers tested. Antibody was detected in two heifers: one from vaccine Gr 4-A and another from control Gr 4-C. These heifers were removed from tables and statistical analyses. All challenged vaccinated heifers in Trial #4 that were sero-negative at 0 DPV had seroconverted by the time of challenge (146/181 DPV; n = 24) with titers ≥1600; all 10 challenged placebo controls (Gr 4-C) remained sero-negative.

Statistical analysis was conducted to determine if there was an association between the presence of *P. abortibovis* antibody and disease protection. All EBA losses in Trial #3 and #4 were from dams that were sero-negative at the time of challenge (i.e., placebo controls; n = 29) and no EBA losses were observed in sero-positive animals (i.e., vaccinated; n = 38). A total of 23 EBA losses were noted in sero-negative dams with the remaining six sero-negative animals either experiencing an undetermined loss or giving birth to a healthy calf. The predictive value that a sero-positive animal will not experience an EBA loss following *P. abortibovis* challenge was 100% (95% CI: 0.8569 to 1.000), whereas the predictive value that a sero-negative animal will experience an EBA loss following bacterial challenge was 86.4% (95% CI: 0.7329 to 0.9360); both were significant (*p* < 0.0001).

## 4. Discussion

Data demonstrates the EBAA vaccine PC #1544.00 poses little to no risk on general animal wellbeing. No incidence of immediate adverse effects, including anaphylaxis, were observed in the 155 cattle administered vaccine, nor was there evidence that vaccination compromised general health within 3 months post-vaccination. There were two incidences of lameness reported throughout the six studies. Lameness can be induced by a variety of known etiologies and is a common malady in feedlot settings [30]. Testing was not conducted to exclude *P. abortibovis* as a cause, however, this pathogen has never been associated with any disease condition in immunocompetent animals [7,9,16] and therefore it is unlikely these illnesses were associated with either EBAA vaccination or *P. abortibovis* challenge.

Vaccine injection site reactions were not anticipated as EBAA vaccine PC #1544.00 does not contain adjuvant and is thus similar to *P. abortibovis*-infected challenge inocula. Overt skin reactions were not noticed in previous infection and immunity studies following inoculation with either *P. abortibovis*-infected bovine or murine challenge inocula [17,18]; studies described here were the first to depilate bacterial injection sites and follow reactions by palpation. Palpable reactions noted in the studies reported here were benign with no evidence of suppuration or irritation. Data indicates that most, if not all, naïve cattle will develop transient injection site reactions (Figure 2), the majority of which will be first detected 20 to 47 days post-exposure (Figure 3) and remain for days to weeks.

Conception rates in these four trials ranged between 80% and 100% regardless of vaccination group and are consistent with the 64% to 95% range expected for first breeding-season pregnancy rates in beef heifers [32]. Rates were similar between vaccinated and unvaccinated animals, with no significant differences noted between groups in any of the 4 studies reported here. However, the vaccine is live and unattenuated; administration to a pregnant animal through the second trimester of gestation, will cause epizootic bovine abortion. Historically, histopathology studies suggest that lesions can develop in fetuses whose dams have been exposed to *P. abortibovis* as late as 200 days of gestation [21]. Further investigations into the safety of late-gestation exposure to the pathogen are underway.

Early fetal losses in cattle are not uncommon and estimated to occur in 5.8% of bovine pregnancies; causes vary but include lethal genetic mutations, placental insufficiency and disease [33]. Therefore, it was not surprising that some early fetal losses were noted amongst the 180 bred heifers in these four reported efficacy trials. The fact that all losses occurred within two groups of vaccinated, subsequently challenged heifers (Gr 2-A and Gr 3-A) caught the attention of researchers. Losses in Gr 2-A (8000 *P.a.*-LIC) comprised 20% of vaccinated, challenged heifers, spanned an 88- to 151-day interval between vaccination and conception, and were divided between heifers artificially inseminated with Angus semen and heifers bred to Brangus bulls by natural service (n = 3 and n = 2, respectively). In comparison, Gr 3-A (2000 *P.a.*-LIC) experienced a 41.7% loss amongst the vaccinated, challenged heifers, all of which were artificially inseminated with Angus semen at 21 DPV. Unlike Gr 2-A, losses in Gr 3-A were significant. Vaccine regimen and vaccination-to-breeding intervals in Gr 2-A were most comparable to Gr 1-A (Table 1), in which no losses were noted. Variables between Gr 3-A (41.7% loss) and Gr 4-A (0% loss) were minimal with the exception of an increased time interval between vaccination and breeding by AI from 21 DPV to 56 DPV, respectively. Analysis limited to the comparison of Gr 3-A and Gr 4-A supports improved vaccine safety if the vaccination-to-breeding interval is extended to ≥56 days. Caution dictates this minimal interval of 56 days be allowed between either inoculation with live EBAA vaccine or pasturing cattle in habitat that supports the tick vector for the purpose of generating natural immunity. Further investigations to better define parameters associated with *P. abortibovis* infection and early fetal loss are underway.

The EBAA vaccine PC #1544.00 proved 100% effective in preventing diagnosed EBA across all four efficacy trials. When prevented fraction calculations incorporated losses in which EBA could not be ruled out (i.e., undetermined losses) vaccine efficacy remained high, ranging from 67% to 100%. Results were statistically significant for all but Trial #1, which lacked significance due to low numbers of data points (Table 3). A strong predictive value (1.0) was identified between the presence of pre-challenge *P. abortibovis* antibody and resistance to live *P. abortibovis* challenge-associated fetal losses.

The *P. abortibovis*-specific IFAT assay described in these studies was reported to be >98% sensitive and >95% specific when diagnostically applied to bovine fetal fluids [29]; a small percentage of fetal fluid samples were believed to contain low-titer cross-reactive antibodies. Sensitivity and specificity of the IFAT assay have yet to be defined for mature animals. Unlike infected bovine fetuses in which changes in histopathology and identification of the organism by either IHC or PCR [13,15,18] can be used to corroborate *P. abortibovis* infection, there is no “gold-standard” to confirm infection in cattle aside from documented EBA abortions. Neither disease associated with *P. abortibovis* infection nor detection of *P. abortibovis* has been reported in immunologically mature animals [9,21]. The University of Nevada MSFL was chosen for these studies based upon the availability of naïve heifers in their beef breeding program and an apparent absence of the tick vector, *O. coriaceus,* in their irrigated pastures. Two of 122 heifer sera collected on day 0 in Trial #3 and #4 were positive for *P. abortibovis* antibodies; both positives were in Trial #4. With no known vector exposure, these results suggested that both were false-positive reactions. However, since the completion of these studies, *O. coriaceus* has been collected along the Truckee River, which is adjacent to some pastures at the UNR-MSFL [34] and increases the probability that antibodies detected in one or both of the sero-positive heifers were specific for *P. abortibovis.*

Prior to these vaccine-directed studies, interactions between *P. abortibovis* and an immunologically mature host had been a black box. The combination of data from injection site reactions, early fetal losses, and vaccine efficacy data pose intriguing insights into *P. abortibovis* pathogenesis. The delayed response at the vaccine injection site suggests a stealth pathogen sequestered within macrophages that does little to activate classic innate immune response cascades associated with inflammation [19,20,35]. It is likely that bacteria and/or bacterial antigens are transported from the initial site of infection to adjacent lymph nodes, initiating a primary immune response. Macrophages and lymphocytes, including plasma cells, were noted in deep muscle biopsies of vaccine injection sites taken in Safety Study #1. These suggest effector cells were attracted back to the injection site by the presence of residual bacterial antigens, resulting in additional immune responses that culminate in a robust amnestic response, thus negating the need for a vaccine booster to confer protection [36,37]. Meanwhile, the remaining live bacteria from the challenge inoculum would have the opportunity to infect resident bovine macrophages and/or as yet undescribed host cells. Detection of *P. abortibovis* in most all fetal organs [19] is reported. The logical conclusion that the bacteria must migrate from the point of infection, through the placenta and to the fetus supports the hypothesis that *P. abortibovis*-infected bovine cells, presumably macrophages, and/or vaccine-derived *P. abortibovis*-infected murine cells, would disseminate to other parts of the body, including the uterus. Data collected on early fetal losses suggest that the incubation period between *P. abortibovis* infection of an embryo and fetal loss is similar to that of a fetus infected later in gestation (i.e., >100 days). Studies reported here were not designed to detect *P. abortibovis* at the injection site nor to study bacterial dissemination; further investigations are needed.

## 5. Conclusions

Data supports EBAA vaccine PC #1544.00 as being safe and effective. Incidence of early fetal losses appeared mitigated if EBAA vaccine was administered at a minimum of 60 days prior to breeding. Efficacy approached 100% for all potencies tested. Seroconversion prior to challenge was predictive for protection of the fetus from epizootic bovine abortion.

## Figures and Tables

**Table 1:** Study design and conception rates.

Trial- Group	Vaccine Regimen (n) ^1^	Interval	% (n) Bred by AI ^3^	% (n) Bred by NS ^4^	Conception Rates		
		Vac-AI (d) ^2^			% (n) ^5^	RR ^6^	95% CI ^7^
1-A	1X-10,000 (5 *)	113	40% (2)	60.0% (3)	100% (5)	0.00	0.000 to 3.299
1-B	2X-10,000 (5)	113/85	25% (1)	75.0% (3)	80% (4)	1.00	0.122 to 8.200
1-C	2X-1000 (6)	37/17	40% (2)	60.0% (3)	83% (5)	0.96	0.432 to 1.950
1-D	None (5)	NA	50% (2)	50.0% (2)	80% (4)	-	-
2-A	1X-8000 (28)	88(An) ^8^/81(Br) ^9^	42.9% (12)	57.1% (16)	100% (28)	0.00	0.000–2.400
2-B	Vac-1X-1000 (7)	63 (An) ^8^	71.4% (5)	28.6% (2)	100% (7)	0.00	0.000 to 8.860
2-C	None (21)	NA	45.0% (9)	55.0% (11)	95% (20)	-	-
3-A	1X-2000 (32)	21	77.8% (21)	22.2% (6)	84.3% (27)	1.67	0.477–5.94
3-B	1X-PBS (32)	NA	58.6% (17)	41.4% (12)	90.6% (29)	-	-
4-A	1X-2000 (20)	56	58.8% (10)	41.2% (7)	85.0% (17)	∞	0.615–∞
4-B	1X-500 (22)	56	60% (12)	40% (8)	90.9% (20)	∞	0.363–∞
4-C	1X-PBS (14)	NA	50.0% (7)	50.0% (7)	100% (14)	-	-

^1^ Denotes dosages (none, 1X or 2X), vaccine potency (# of live, *Pajaroellobacter abortibovis*-infected murine spleen cells) and number of animals per group (n); ^2^ Interval, in days (d), between vaccination and artificial insemination (AI); ^3^ Percentage and number of animals (n) bred by AI; ^4^ Percentage and number of animals (n) bred by natural service; ^5^ Percentage and number (n) of total animals bred; ^6^ Relative risk of failing to conceive if vaccinated; ^7^ 95% Confidence interval in RR values; none were significant (*p* > 0.05); ^8^ Angus sire; ^9^ Brangus sire; NA = Not applicable; * 1 of 6 heifers in Gr 1-A died prior to breeding, leaving 5.

**Table 2:** Early fetal losses: previously pregnant dams that were open 3 to 7 months into gestation.

Trial- Group	Vaccine Regimen	Number Bred	Number Challenged	Early Fetal Loss		
				% (n) ^1^	RR ^2^	95% CI ^3^
1-A	1X-10,000	5	4	0	NA	NA
1-B	2X-10,000	4	4	0	NA	NA
1-C	2X-1000	5	4	0	NA	NA
1-D	Control	4	4	0	-	-
2-A	1X-8000	28	25	20% (5)	∞	0.744–∞ ^ns^
2-B	1X-1000	7	7	0	NA	NA
2-C	Control	20	12	0	-	-
3-A	1X-2000	27	24	41.7% (10)	∞	2.0–∞ ***
3-B	Control	29	20	0	-	-
4-A	1X-2000	17	5	0	NA	NA
4-B	1X-500	20	19	0	NA	NA
4-C	Control	14	9 *	0	NA	NA

^1^ Percentage and number (n) of animals experiencing early fetal losses; ^2^ Relative risk of early fetal loss amongst challenged vaccinates vs. challenged controls; ^3^ 95% Confidence interval in RR values; ns = not statistically significant; *** *p* < 0.001; NA = Not applicable; * 1 of 10 control heifers in Gr 4-C died post-challenge, leaving 9.

**Table 3:** Vaccine efficacy.

Trial- Group/ Regimen	Number Preg ^1^	% (n) EBA Losses ^2^	% (n) EBA Und ^3^	% (n) Non-EBA ^4^	EBA Losses		EBA/UND	
					PF ^5^	95% CI ^6^	PF ^5^	95% CI ^6^
1-A	4	0% (0)	0% (0)	100% (4)	1.00	−0.390–1.0 ^ns^	1.00	−0.390–1.0 ^ns^
1X-10,000	(2/2)	(0/0)	(0/0)	(2/2)	(1.0/1.0)	(−1.68–1.0/−1.68–1.0)	(1.0/1.0)	(−1.68–1.0/−1.68–1.0)
1-B	4	0% (0)	0% (0)	100% (4)	1.00	−0.390–1.0 ^ns^	1.00	−0.390–1.0 ^ns^
2X-10,000	(2/2)	(0/0)	(0/0)	(2/2)	(1.0/1.0)	Same as 1-A	(1.0/1.0)	Same as 1-A
1-C	4	0% (0)	0% (0)	100% (4)	1.00	−0.390–1.0 ^ns^	1.00	−0.390–1.0 ^ns^
2X-1000	(2/2)	(0/0)	(0/0)	(2/2)	(1.0/1.0)	Same as 1-A	(1.0/1.0)	Same as 1-A
1-D	4	50% (2)	0% (0)	50% (2)	-	-	-	-
Control	(2/2)	(1/1)	(0/0)	(1/1)	-	-	-	-
2-A	20	0% (0)	15% (3)	85% (17)	1.00	0.781–1.0 ****	0.84	0.591–0.943 ****
1X-8000	(10/10)	(0/0)	(1/2)	(9/8)	(1.0/1.0)	(0.72–1.0 ***/0.36–1.0 *)	(0.90/0.76)	(0.60–0.98 ***/0.25–0.93 *)
2-B	7	0% (0)	0% (0)	100% (7)	1.00	0.551–1.0 **	1.00	0.608–1.0 ***
1X-1000	(7/NA)	(0/NA)	(0/NA)	(7/NA)	(1.0/NA)	(0.65–1.0 ***/NA)	(1.0/NA)	(0.65–1.0 ***/NA)
2-C	12	75% (9)	16.7% (2)	8.3% (1)			-	-
Control	(6/6)	(6/3)	(0/2)	(0/1)			-	-
3-A	14	0% (0)	21.5% (3)	78.5% (11)	1.00	0.686–1.0 ****	0.67	0.319–0.901**
1X-2000	(11/3)	(0/0)	(2/1)	(9/2)	(1.0/1.0)	(0.59–1.0 ***/0.36–1.0 ^ns^)	(0.74/0.67)	(0.26–0.93*/−0.190–0.39 ^ns^)
3-B	20	70% (14)	5% (1)	25% (5)	-	-	-	-
Control	(17/3)	(11/3)	(1/0)	(5/0)	-	-	-	-
4-A	5	0% (0)	0% (0)	100% (5)	1.00	0.566–1.0 ***	1.00	0.566–1.0 ***
1X-2000	(5/NA)	(0/NA)	(0/NA)	(5/NA)	(1.0/NA)	(0.57–1.0 **/NA)	(1.0/NA)	(0.57–1.0 **/NA)
4-B	19	0% (0)	5.2% (1)	94.7% (18)	1.00	0.832–1.0 ****	0.95	0.754–0.991 ****
1X-500	(12/7)	(0/0)	(1/0)	(11/7)	(1.0/1.0)	(0.758–1.0 ****/0.616–1.0 **)	(0.92/1.0)	(0.646–0.985 ***/0.616–1.0 **)
4-C	9	100% (9)	0% (0)	0% (0)	-	-	-	-
Control	(6/3)	(6/3)	(0/0)	(0/0)	-	-	-	-

^1^ Number of challenged dams that remained pregnant at >7 months of gestation; numbers in parentheses indicate data for challenge groups 1 and 2, respectively; ^2^ Fetuses/neonates with diagnosis of EBA; ^3^ Fetuses/neonates whose EBA status was undetermined (Und); ^4^ Fetuses/neonates that were either born healthy or died from a cause other than EBA; ^5^ Prevented fraction; ^6^ 95% Confidence interval for PF values; NA = Not applicable; ns = not statistically significant, * *p* < 0.05; ** *p* < 0.01; *** *p* <0.001; **** *p* ≤ 0.0001.

## Data Availability

Contact corresponding authors for data requests.

## Supplementary Materials

The following supporting information can be downloaded at: https://www.mdpi.com/article/10.3390/vaccines10020335/s1. Figure S1: Timelines for Efficacy Trials #1–4.
