# Supplementary material for: Protection of Cattle against Epizootic Bovine Abortion (EBA) Using a Live Pajaroellobacter abortibovis Vaccine
Source: Vaccines (Basel). 2022 Feb 19;10(2):335. doi: 10.3390/vaccines10020335 (PMC8874702; doi:10.3390/vaccines10020335)
Supplement: Supplementary file 1 [file vaccines-10-00335-s001.zip › vaccines-1559467-Supplementary material.pdf]

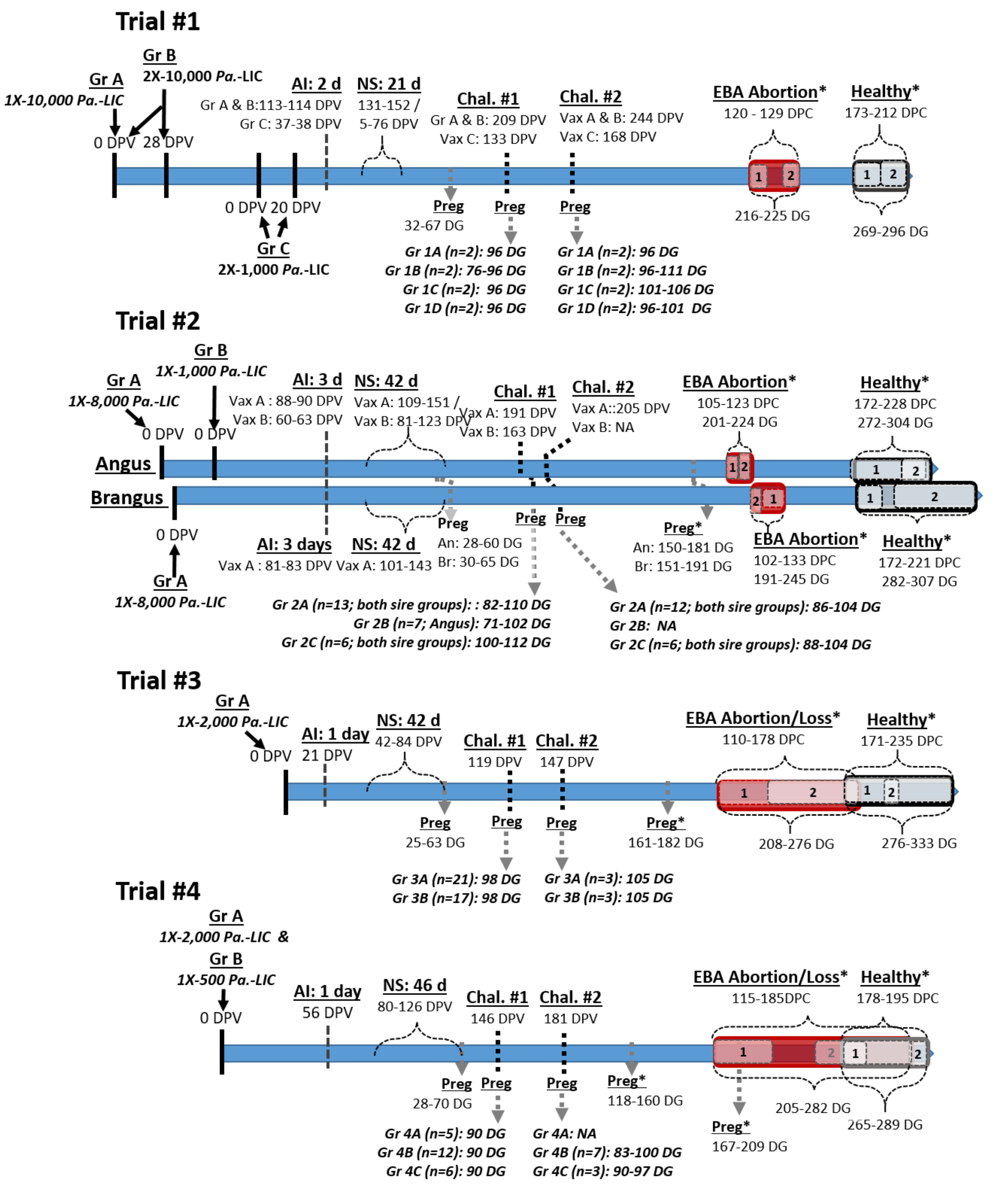

Figure S1. Experimental timeline of vaccination, breeding by either artificial insemination (AI) or natural bull service (NS), pregnancy examinations (Preg), *Pajaroellobacter abortibovis* challenge (Chal), abortions/calf losses and healthy births for each of 4 efficacy trials. Temporal references include days post-vaccination (DPV), days post-challenge (DPC) and days fetal gestation (DG). Abortions due to EBA (EBA Abortion), losses from other causes (Losses) and full-term healthy calves (Healthy) are noted for challenged animals only(*). Boxes within "EBA Abortions/Losses" and "Healthy" calving illustrate the timing of these events in challenge groups 1 vs. 2. Trial timelines are to relative scale and aligned at time of artificial insemination (AI) for better visual reference to varying intervals between vaccination and breeding.
